# The Microbial Diversity and Flavor Metabolism Regulation of *Xiangzao* During Different Natural Fermentation Time Periods

**DOI:** 10.3390/foods13233931

**Published:** 2024-12-05

**Authors:** Rongbin Zhang, Shuangping Liu, Tiantian Liu, Rui Chang, Guixiao Liu, Mingliang Li, Jian Mao

**Affiliations:** 1 National Engineering Research Center of Cereal Fermentation and Food Biomanufacturing, State Key Laboratory of Food Science and Technology, School of Food Science and Technology, Jiangnan University, Wuxi 214122, China; zhangrb@ms.giec.ac.cn (R.Z.); liushuangping668@126.com (S.L.); 7200112052@stu.jiangnan.edu.cn (R.C.); lml13715@163.com (M.L.); 2 Guangzhou Institute of Energy Conversion, Chinese Academy of Sciences, Guangzhou 510630, China; 3 Shaoxing Key Laboratory of Traditional Fermentation Food and Human Health, Jiangnan University (Shaoxing) Industrial Technology Research Institute, Shaoxing 312000, China; lgx13205213195@163.com

**Keywords:** *Xiangzao* brine, microbiome, flavor substances, umami peptide, metabolic mechanism

## Abstract

*Xiangzao* brine is a special flavored food produced by the natural fermentation of *Huangjiu* lees. To clarify fermentation time on its quality, this study integrated flavoromics analysis, macro-genomics, and polypeptide omics to analyze the volatile flavor components, microbial species, and flavor peptide distributions of four groups of samples (XZ-1Y, XZ-2Y, XZ-3Y, and XZ-4Y) fermented for 1–4 years. The results showed that the samples fermented for 1 year had the highest contents of umami amino acids and umami peptides, and the samples fermented for 4 years had the highest contents of organic acids and fruity components. In addition, 42 volatile flavor components and 532 peptides were identified, including 393 umami taste peptides and only 37 bitter taste peptides. Correlation analysis showed that ethyl lactate and furfural were positively correlated with the abundance of *Nocardioides* and *Stenotrophomonas*, respectively. The abundance of *Pseudomonas* was positively correlated with four previously unreported umami peptides (FATPR, RELER, FNLERP, and RSSFLGQ) screened by molecular docking. This study provides a reference for the flavor metabolism regulation of *Xiangzao* brine.

## 1. Introduction

Huangjiu is one of the most popular fermented foods in the world [1]. It is brewed from rice, wheat qu, and yeast, and it contains more than 600 volatile and nearly 1000 non-volatile flavor components [2]. The solid residue produced by extrusion and filtration at the end of the brewing process is called *Huangjiu* lees, with up to 3.5 million tons produced annually. *Huangjiu* lees contain a large number of nutrients, including protein, fat, cellulose, vitamins, minerals, etc. Direct disposal of distillers’ grain not only pollutes the environment but also causes a waste of resources. Common disposal strategies include feed, fertilizer, culture medium of edible fungus, etc., but the added value is low [3]. Recent studies from our research group have found that the enzymatic transformation of *Huangjiu* lees can produce umami peptides, indicating that it has the potential to increase flavor value. The lees through brewing still keeps a part of the flavor components and a large number of microorganisms [4]. Therefore, *Huangjiu* lees can be a natural source of flavor enhancers through rational fermentation and utilization.

In fact, Chinese people used *Huangjiu* lees to improve food flavor by taking advantage of its unique flavor and its ability to be fermented twice thousands of years ago, but the industrial scale was small [5]. *Xiangzao* brine is popular with consumers in southern China. It is made from aged fermented *Huangjiu* lees by extracting lees juice through a special process and refined with a spicy sauce. The characteristics of *Xiangzao* brine are transparent color without sediment and a strong lees aroma, which can effectively remove fishy and muttony taste and enhance the favor of food. The use of *Huangjiu* lees in the preparation of *Xiangzao* brine not only inherits the traditional Chinese brewing process but also provides a new way for the comprehensive utilization of *Huangjiu* lees [6]. However, at present, the preparation of *Xiangzao* brine is carried out in a relatively uncontrolled and self-fermenting way resulting of unstable flavor and quality, which cannot be produced in batches. Therefore, it is necessary to understand its relationship between the microbial composition and the regulatory pathways of flavor substances.

For fermented food, microbial activities play a key role in the formation of metabolites and flavors. Traditional methods to analyze them mainly rely on in vitro culture, isolation, purification, and identification, which are less efficient. The development of food genomics technologies has greatly improved the identification efficiency of microorganisms and products, including LC-MS/MS-based metabolomics, GC-MS/MS-based flavoromics, and 16s-rRNA-sequencing-based microbiomics. However, relying on only a certain type of omics data cannot clarify which microorganisms perform the targeted metabolic activities during fermentation [7,8]. Combined multi-omics analysis can provide insights into the correlation between the metabolic pathways of flavor substance and microbial community structure [9,10]. For example, Wu et al. [10] constructed a metabolic network of characteristic flavor components in vinegar and analyzed the key microorganisms therein through metagenomics. Liu et al. [11] conducted a study on the fermentation of *Huangjiu* using multi-omics techniques and found that *Saccharopolyspora* as the dominant functional genus in wheat yeast was responsible for the decomposition of starch and cellulose. Lan et al. [12] found that *Staphylococcus* dominates the fermentation products of douchi (fermented beans) and is positively correlated with the 1-octene-3-ol content of mushroom flavor. For *Xiangzao* brine, previous studies have focused on its microbial community analysis [13] or volatile flavor analysis [14], but the correlation between flavor peptide, flavor components, and microorganisms are unclear.

This study examined a 1–4 year storage period to find the best time for optimal flavor development at the lowest cost, as traditional fragrant grains are usually stored for 1–3 years. First, the physicochemical indexes and changes of flavor substances in different fermentation years (XZ-1Y, XZ-2Y, XZ-3Y, XZ-4Y) of *Xiangzao* made of *Huangjiu* lees were detected. Then, multi-omics techniques (flavoromics, microbiomics, and peptideomics) were integrated to reveal the dynamic changes and correlations of volatile flavor substances, flavor peptides, and the bacteria in the fermentation process of *Xiangzao* brine. In addition, the conformational relationships of the flavor peptides were discussed through the molecular docking. The aim of this study was to provide scientific support for improving the fermentation flavor quality of *Xiangzao* and provide a theoretical basis for its quality control.

## 2. Materials and Methods

### 2.1. Experimental Materials

*Xiangzao* (XZ-1Y, XZ-2Y, XZ-3Y, XZ-4Y) of 1–4 fermentation years was provided by Jinfeng Wine Company Limited, Shanghai China. Sodium hydroxide, hydrochloric acid, glacial acetic acid, and other analytical reagents were bought from Sinopharm Chemical Reagent Co., LTD, Shanghai China. Colorimetric-grade acetonitrile was purchased from Titan technology co., LTD, Shanghai China. Amino acid standards were purchased from Anpel Experimental Technology Co., LTD, Shanghai China.

### 2.2. Experiment Method

#### 2.2.1. Determination of Physicochemical Indexes of *Xiangzao*

Sample pretreatment: 10 g of solid sample was weighed and placed in a 250 mL beaker. A total of 100 mL of 0.9% sodium chloride solution was also added, and the mixture was stirred well. The sample was soaked for 30 min at room temperature with frequent stirring. After being filtered through filter paper, the sample was placed on standby for determination. The determination of pH value, total acidity, and amino acid nitrogen was conducted in accordance with the national standard GB/T13662-2018 Sections 6.4, 6.5, and 6.6 respectively [15]. The HPLC method [6,16] was used to determine the content of organic acids in *Xiangzao*. The determination of total nitrogen used the Kjeldahl methods [17].

#### 2.2.2. Determination of Volatile Flavor Components of *Xiangzao*

The HS-SPME/GC-MS method [18,19] was used to determine the volatile flavor compounds in *Huangjiu*.

#### 2.2.3. Analysis of Flavor Peptides of *Xiangzao*

##### Identification of Peptides of *Xiangzao*

The polypeptide composition of *Xiangzao* was identified by label-free quantitative (LFQ) polypeptidomics, and the method was in reference to Chang et al. [20]. The sample to be tested was treated with lysate (8M urea, 1x protease inhibitor cocktail), and the supernatant was taken after treatment. Supernatant fluid was desalinated by MonoSpin C18 and was analyzed by LC-MS/MS equipped with an on-line nanoparticles source. The mass spectrometer runs in data-dependent acquisition mode, automatically switching between MS and MS/MS acquisition. The mass spectrometry parameters were set as follows: (1) MS: scan range (*m*/*z*): 350–1500, resolution: 120,000; AGC target: 8 × 10^5^, maximum injection time: 50 ms. (2) HCD-MS/MS: resolution: 30,000, AGC target: 1 × 10^5^, maximum injection time: 54 ms; collision energy: 25,30,35; dynamic exclusion time: 30 s. The tandem mass spectrogram was analyzed by PEAKS Studio version 10.6. Set No enzyme to be non-enzyme digestion. According to Uniprot and NCBI databases, we built a protein library and performed qualitative and quantitative analysis of peptides according to the search results.

##### Prediction of the Flavor Peptides of *Xiangzao* Samples

The identified peptide sequences were used to predict the flavor potential. Umami_YYDS2.0 and TastePeptidesDM online tools (http://www.tastepeptides-meta.com accessed on 22 June 2024) were used for taste prediction. Bitterness was predicted by using Umami_YYDS2.0 and BERT4Bitter online tools (http://pmlab.pythonanywhere.com/BERT4Bitter accessed on 22 June 2024). The peptides screened by the two tools together were used as potential flavor peptides [21,22]. In addition, the physicochemical properties of peptides, including isoelectric points, mean hydrophilicity values, and molecular weights, were analyzed.

##### Screening of Umami Peptides of *Xiangzao* Samples

Molecular docking screening was performed with high abundance of differentially flavored peptides common to different samples. The peptide structures were mapped by Chemdraw program and energy minimized by MMFF94 force field. The metabotropic glutamate receptor (PDB: 6N51) with high sequence similarity was used as the homologous reference template for human-derived umami receptors T1R1-T1R3. The Autodock Vina1.2.3 program was used for docking, and the docking parameters were in reference to Zhang et al. [23].

#### 2.2.4. Microbiome Analysis of *Xiangzao* Samples

Full-length 16s amplicon sequencing was performed based on the PacBio sequencing platform. The samples were extracted by CTAB, and the libraries were sequenced by PacBio Sequel II. The Raw-CCS sequence data were identified by barcode using lima v1.7.0 software. The Clean-CCS sequence without primer sequence was obtained using the cutadapt 1.9.1 software. The Effective-CCS sequence was obtained by identifying and removing the chimeric sequences using UCHIME v4.2 software. Finally, high-quality sequences were obtained for subsequent omics analysis.

#### 2.2.5. Correlation Analysis Between Microbial Diversity and Flavor Components in *Xiangzao*

The Pearson correlation coefficient method was used to analyze the correlation between the key volatile flavor components and flavor peptides.

#### 2.2.6. Methods of Analysis

All experiments were performed in triplicate, and the data were processed and visualized using Origin 2024. One-way ANOVA analysis of variance was performed using SPSS Statistics 27, with the statistical significance level set at *p* < 0.05. Usearch 10.0.240_i86 software was used to cluster reads at a 97.0% similarity level to obtain OTU. Samples were evaluated using QIIME2 2020.6.0 software for the four α diversity indexes. Species composition analysis and visualization were performed by the BMKClou platform (www.biocloud.net). The biomarker with statistically significant differences between groups was analyzed using the Python LEfse package. Fungi Functional Guild 1.0 was used to predict fungal phenotypes. STAMP software was used to test the significant differences between any two samples with *p*-value < 0.05 indicating significance. Intersample partial least squares (PLS-DA) analysis was performed on the abundance of the peptides by using the SIMCA14.1 program.

## 3. Results

### 3.1. Analysis of Physicochemical Indexes of Xiangzao in Different Fermentation Years

#### 3.1.1. Analysis of pH Value and Total Acid and Organic Acid Content of *Xiangzao* in Different Fermentation Years

**Figure 1:**
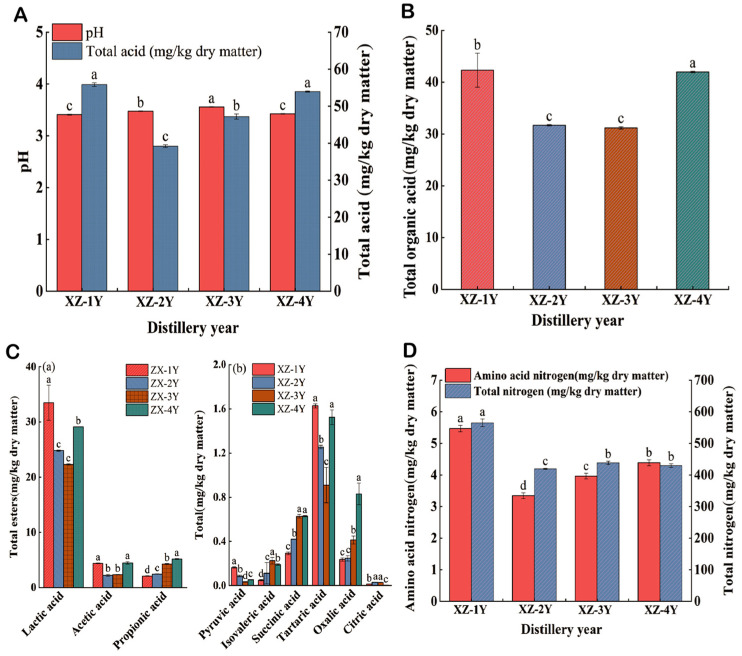
Physicochemical indices of *Xiangzao* in different fermentation years. (**A**) pH and total acid. (**B**) Total organic acids. (**C**) The number of different kinds of organic acids, C(a); major organic acids, C(b); other organic acids. (**D**) Amino acid nitrogen and total nitrogen. Note: (a–c) Within the same column of data, identical letters indicate no significant difference (*p* > 0.05), while different letters signify a significant difference (*p* < 0.05).

This study analyzed the pH value and total acid of *Xiangzao* in the four years. The results are shown in Figure 1A, which shows that the pH value of *Xiangzao* in four years did not have significant differences between 3.41 and 3.55, but the differences of total acid were obvious. The total acid content of the samples in XZ-2Y was the lowest. The specific data of *Xiangzao* physicochemical indices in different fermentation years are presented in Table S1. 

Organic acids can significantly affect the flavor balance, chemical stability, pH value, and quality of foods [24]. The content of total organic acids in *Xiangzao* of four years is shown in Figure 1B, from which it can be seen that XZ-4Y fermented for four years had the highest content of organic acids, 42.0 mg/kg. However, the organic acid content showed a decreasing trend within 1–3 years of fermentation, with the lowest content in XZ-3Y (31.2 mg/kg dry matter). It indicates that the fermentation process of *Xiangzao* may have stages. As can be seen from Figure 1C, lactic acid content in the XZ-1Y (33.5 mg/kg of dry matter) was significantly higher than XZ-4Y, showing that lactic acid was partially consumed during fermentation. Other organic acids with higher content included acetic acid and propionic acid, and propionic acid gradually increased with the fermentation time.

#### 3.1.2. Analysis of Total Nitrogen and Amino Acid Nitrogen Content in *Xiangzao* in Different Fermentation Years

Figure 1D shows the differences between amino acid nitrogen and total nitrogen in *Xiangzao* for the different years. It also can be seen that the total nitrogen content of *Xiangzao* in four years was significantly higher than that of amino acid nitrogen, which indicated that nitrogen metabolism was low. The contents of amino acid nitrogen and total nitrogen in XZ-1Y were the highest, 5.47 mg/kg dry matter and 565.1 mg/kg dry matter, respectively.

### 3.2. Analysis of Volatile Flavor Substance Content in Xiangzao in Different Fermentation Years

**Figure 2:**
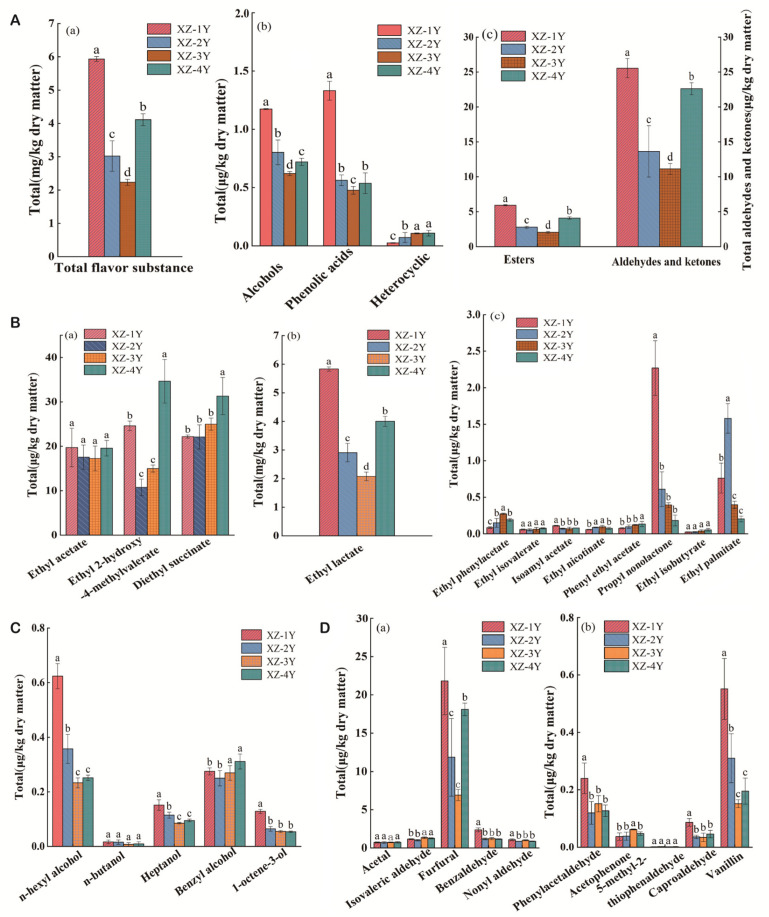
Flavor substances in *Xiangzao* in different fermentation years. (**A**) Total change. A(a) Total flavor substance; A(b), total alcohols, phenolic acids, and heterocyclics; A(c), total esters, aldehydes, and ketones. (**B**) Esters in *Xiangzao* of different fermentation years. (**C**), Alcohols in the halogen of *Xiangzao* in different years. (**D**), Aldehydes and ketones in *Xiangzao* in different years. Note: (a–d) Within the same column of data, identical letters indicate no significant difference (*p* > 0.05), while different letters signify a significant difference (*p* < 0.05).

**Figure 3:**
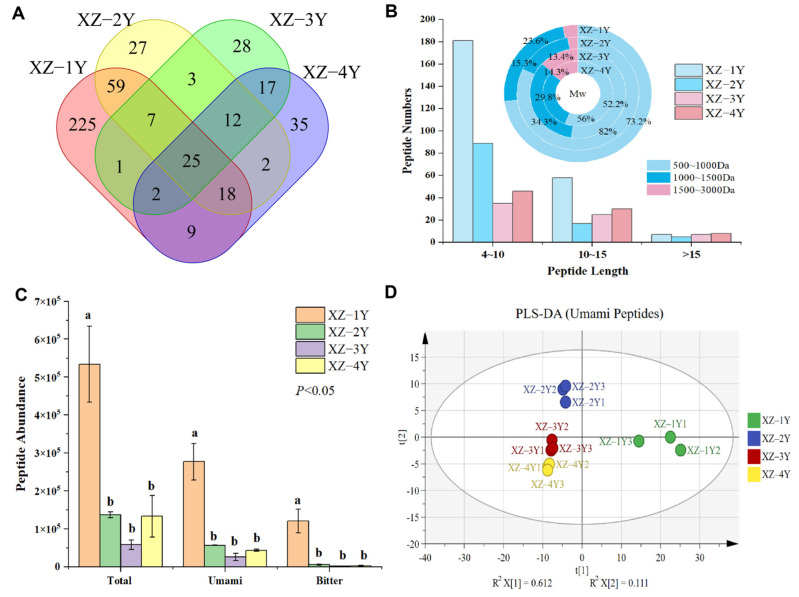
Distribution of umami peptides in different fermentation years. (**A**) Venn diagram of peptide quantity distribution of sample. (**B**) Molecular weight distribution of peptide. (**C**) Peptide abundance in different samples. (**D**) PLS-DA model classification of umami peptides from different samples. Note: (a–b) Within the same column of data, identical letters indicate no significant difference (*p* > 0.05), while different letters signify a significant difference (*p* < 0.05).

Forty-two volatile flavor compounds were quantitatively analyzed in different fermentation years. Figure 2A shows that the total content of flavor substances in XZ-1Y fermented for 1 year was the highest, while that in XZ-3Y fermented for 3 years was the lowest. It was found that the proportion of ester substances was similar to that in *Huangjiu* according to the detection of ester substances in *Xiangzao* (Figure 2B). Therefore, it is possible that the esters in *Xiangzao* are residuals of *Huangjiu* lees [25]. The highest content of ester substances in *Huangjiu* was ethyl lactate (fatty aroma), followed by ethyl acetate (fruity aroma) [26] and diethyl succinate (trace fruity aroma) [27]. Ethyl lactate was highest in XZ-1Y and lowest in XZ-3Y. As can be seen from Figure 2B, 2-hydroxy-4-methylpentanoate (fruity aroma) [28] and diethyl succinate had the highest content in *Xiangzao* of XZ-4Y and the lowest content in XZ-3Y and XZ-2Y, while ethyl acetate had comparable content in the four years of *Xiangzao*. Figure 3B shows that the content of ethyl lactate was the highest in XZ-1Y and the lowest content in XZ-3Y. Figure 2B also shows that the contents of ethyl phenylacetate (honey aroma), propylnonyl lactone (coconut aroma), and ethyl palmitate (milk aroma) were significantly different among the four years, with the highest contents of propylnonyl lactone in XZ-1Y and ethyl palmitate in XZ-2Y, which contributed to the distinctive flavor of *Xiangzao* in different years [29].

Alcohols in *Xiangzao* can greatly improve the layering of dishes, giving them a more mellow taste and unique flavor [30]. The content of alcohols is shown in Figure 2C. The diagram shows that n-hexanol had the highest content and n-butyl alcohol had the lowest content in *Xiangzao*. The content of n-hexanol was the highest in XZ-1Y, followed by XZ-2Y. The content of aldehydes and ketones in *Xiangzao* of the four years is shown in Figure 2D. Furfural had the highest content in the 4 years, while 5-methyl-2-thiophenecarboxaldehyde was the lowest. As can be seen in Figure 2D, the content of furfural was the highest in XZ-1Y and the lowest in XZ-3Y. There was no significant difference in the content of acetaldehyde, isovaleraldehyde, benzaldehyde, and nonaldehyde in *Xiangzao* of the four years. Figure 2D shows that the contents of vanillin, phenylacetaldehyde, and hexanal were the highest in XZ-1Y. In the process of food fermentation, microbial activities not only influence the formation of volatile flavor components but also change the types and proportions of non-volatile components. The protein analysis of the natural fermentation process of *Huangjiu* lees by Liu et al. [31] showed that the microbial activity promoted the accumulation of proteolytic products such as peptides and amino acids. Similarly, *Xiangzao* contains rich protein, and it may produce a large number of low-molecular-weight peptides during fermentation. The specific data of *Xiangzao* volatile flavor compounds in different fermentation years are presented in Supplementary Table S2. 

### 3.3. Analysis of Flavor Peptide of Xiangzao in Different Fermentation Years

#### 3.3.1. The Flavor Peptide Distribution of *Xiangzao*

Low-molecular-weight peptides present potential bioactivity of flavor-presenting characteristics due to their complex amino acid composition, space configuration, and receptor recognition specificity. Peptides with umami flavors have become a new generation of umami agents that are widely found in fermented soybean, meat, and fish, among others [32]. A total of 523 peptides were identified from four groups of *Xiangzao* samples in different years (Figure 3A). In total, 393 peptides with potential umami flavor and 37 bitter peptides were found after prediction by machine learning models. It can be seen that the identified flavor peptides were mainly umami peptides, accounting for 73.87% of the total number of peptides. In samples XZ-1Y, XZ-2Y, XZ-3Y, and XZ-4Y from different fermentation years, 246, 111, 67, and 84 umami peptides were identified, while the numbers of bitter peptides were only 30, 9, 6, and 9, respectively. This showed that the peptides in the samples were mainly umami peptides, which accounted for 70.00~72.54% of the total number of peptides, while the bitter peptides only accounted for 5.88~8.67%.

Molecular weight size affects the potential activity of peptides. Usually, low-molecular-weight peptides are absorbed and metabolized more efficiently [33]. The results in Figure 3B show that the identified flavor peptides were mainly 4–10 peptides, and more than 95% of the peptides in each group had molecular weights less than 1500 da. The proportion of 4–10 peptides in the XZ-3Y group was 52.24%, close to that of the XZ-4Y, which was 54.76%. The proportion of 4–10 peptides in the XZ-1Y and XZ-2Y groups was higher, which amounted to 73.58% and 80.18%, respectively. The XZ-1Y and XZ-2Y groups were 23.48% higher than the other two groups on average as for peptides with molecular weights in the range of 500–1000 Da. However, the proportion of peptides with molecular weights above 1000 Da was much lower than that of XZ-3Y and XZ-4Y groups. The average of the former two was 22.42%, and the average of the latter two was 45.9%. This suggests that the low-molecular-weight peptides were degraded as the fermentation proceeded, and the large molecular proteins in the raw material were gradually cut into long-chain peptides.

Figure 3C shows that the highest peptide abundance was found in the XZ-1Y sample, which showed a decreasing trend with the increase in fermentation time. The abundance of umami peptides was significantly higher than that of bitter peptides, accounting for 32.95–51.87% of the total abundance, and the XZ-1Y sample was the most abundant. Meanwhile, the highest abundance of bitter peptides was observed in the XZ-1Y sample (22.56%), which was much larger than that of the other samples (2.32~4.47%). This indicated that bitter peptides decreased significantly after long fermentation. The PLS-DA classification results of the umami peptides in *Xiangzao* samples in different years also show that XZ-3Y and XZ-4Y samples were similar (Figure 3D).

The abundance of aromatic distiller polypeptides in different years was compared and analyzed (Figure S1). A total of 199, 209, and 181 peptides were differentially down-regulated between the XZ-1Y sample and other samples of different years (XZ-2Y, XZ-3Y, and XZ-4Y), respectively. Only 25, 17, and 25 peptides were up-regulated, respectively. The differentially down-regulated peptides were mainly umami peptides, and 141, 147, and 129 peptides were down-regulated between the XZ-1Y sample and other samples from different years, respectively. A total of 124 differentially down-regulated umami peptides were found among the samples of each group. A total of 25 peptides were less than 10 in length and more than 1% in average abundance, which might be the main source of umami peptides in *Xiangzao*.

#### 3.3.2. Screening of Umami Peptides from *Xiangzao*

The 25 differential umami peptides with high abundance were further screened by molecular docking, the results are shown in Table S3. The flavor peptides were screened based on peptide length less than 10 and binding energy less than -8 kcal/mol to the receptor T1R1. Eight umami peptides, RATPR, RELER, FNLERP, TYNPR, DSSNPR, RSSFLGQ, AGAPAHS, and VCGLVHDAGG, were selected, which were, respectively, from *Saccharomyces cerevisiae* unknown function protein domain, *Saccharomyces cerevisiae* non-ribosomal peptide synthetase, Antarctic fur acetyl amino transferase homoserine bacterium, millet seed protein subunits, rice Golgi complex subunits, Antarctic fur pre-benzoic acid bacterium dehydrogenase, *Saccharomyces cerevisiae*, and glycine dehydrogenase. TYNPR is a reported umami peptide in *Huangjiu* with an umami taste threshold of 2.11 mmol/L [34]. The analysis of the precursor proteins of the eight umami peptides showed that they were mainly derived from rice and yeast. Traceability analysis showed that microbial-derived peptides are important components of flavor peptides. Yeast releases potential active peptides through autolysis during fermentation [35]. Other microorganisms may participate in the formation of peptides through metabolic pathways.

**Figure 4:**
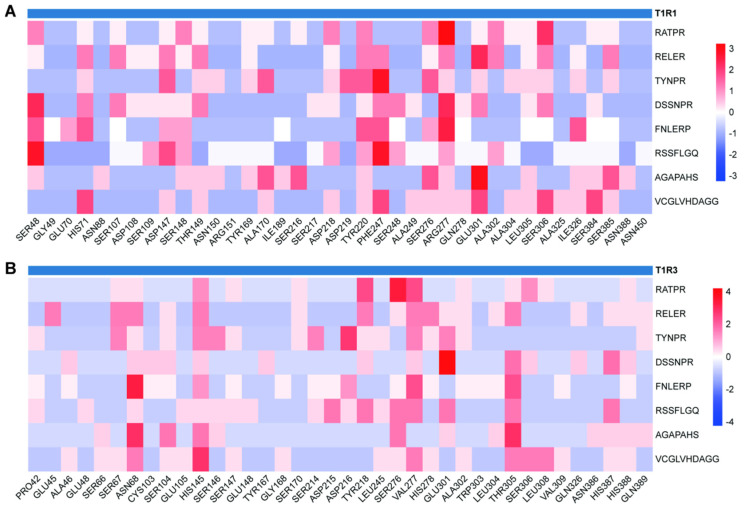
Heat map of interaction between *Xiangzao* umami peptide and T1R1/T1R3 receptor residues. (**A**), Umami peptide interacts with T1R1. (**B**), Umami peptide interacts with T1R3.

Residue interaction results showed that SER48, HIS71, ASP147, TYR220, PHE247, ARG277, GLU301, and SER306 were the main residues of T1R1 receptor interacting with the umami taste peptide (Figure 4A). SER48, ARG277, and ASP147 were mainly involved in the formation of hydrogen bonding. For the T1R3 receptor, ASN68, HIS145, VAL277, and THR305 were the main interacting residues. ASN68, HIS145, and THR305 were mainly involved in the formation of hydrogen bonds. Studies on the peptides in the hydrolysates of porcine collagen showed that the above residues as well as ASN68, HIS71, ASP147, ARG277, VAL277, THR305, SER306, and TYR220 were key residues in the interaction between umami peptides such as CN, SM, CRD, and GESMTDGF and T1R1/T1R3 receptors [36]. SER48 could not be ignored in the interaction between umami peptides Boletus edulis DPY, TEWH, YDKL, HHYE, and umami receptors [4]. Four previously unreported peptides, FATPR, RELER, FNLERP, and RSSFLGQ, with strong umami potential were screened by combining the bond forming type and residue binding effect. All of them were peptides with low binding energy to the receptor, indicating the contribution of key interacting residues in the formation of binding energy. The free energy decomposition of umami peptides with active pocket residues of the receptor by Zhang et al. [37] also showed the dominance of hydrogen bonding interactions. Binding pattern analysis plots performed on four peptides with strong umami potential showed (Figure 4B) that all four peptides were located in the umami receptor Venus flytrap domain. The weak molecular force generated by the large number of hydrophobic residues contacted by the peptides around this region played an important role in stabilizing the conformation [38]. The above analysis suggested that umami peptide of *Xiangzao* might exert umami taste by interacting with the ligand-binding domain of the umami receptor.

### 3.4. Microbiome Analysis of Xiangzao in Different Years

#### Analysis of Microbial Community Structure in Xiangzao in Different Years

**Figure 5:**
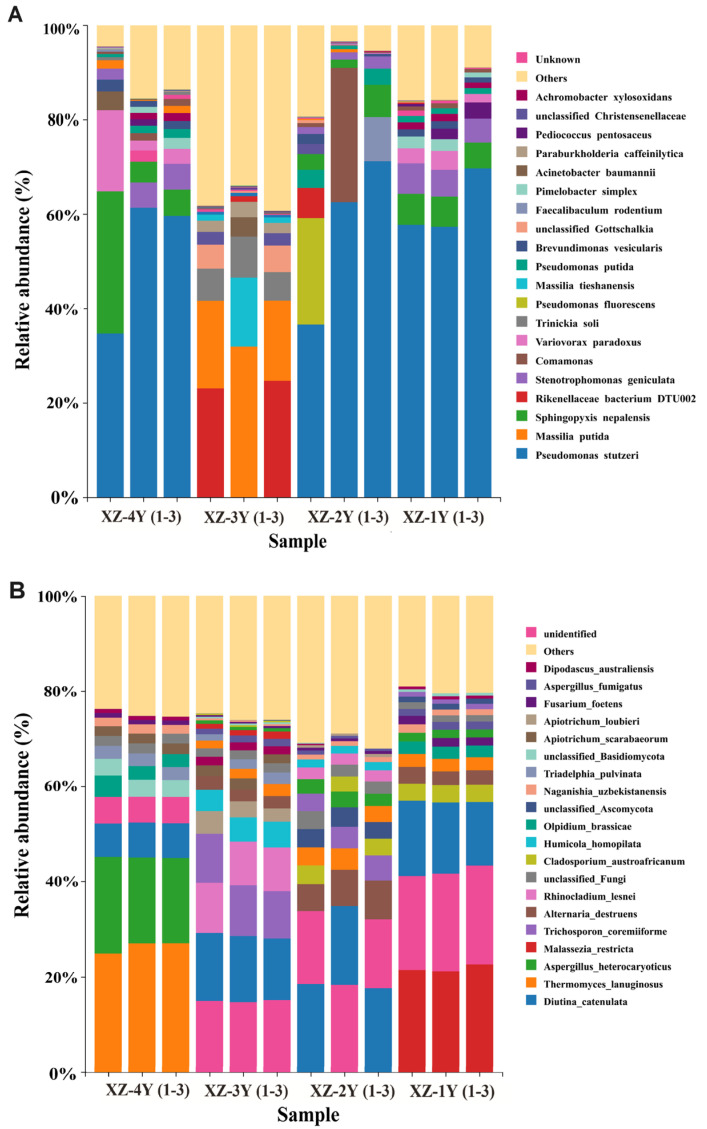
The microbial community structure of *Xiangzao*. (**A**) Bacteria; (**B**) fungi. (1–3) Three parallel data are represented.

As can be seen in Figure 5A, for the bacterial community, the top ten of each sample in terms of abundance were Pseudomonas stutzeri, Massilia putida Sphingopyxis nepalensis, Rikenellaceae bacterium DTU002 Stenotrophomonas geniculata, Comamonas Variovorax paradoxus, Trinickia soli, Pseudomonas fluorescens, Massilia tieshanensis, and Pseudomonas putida. For the fungal community (Figure 5B), the abundance of the top ten of each sample were Thermomyces lanuginosus, Diutina catenulata, Hydrophobic spongiform thermophilic mildew, Aspergillus heterocaryoticus, Malassezia restricta, Trichosporon coremiiforme, Alternaria destruens, Rhinocladium lesnei, unclassified Fungi, Cladosporium austroafricanum, Humicola homopilata, and Olpidium brassicae. The composition of bacterial and fungal microbial communities in Xiangzao, are meticulously detailed in Tables S4 and S5, respectively.

### 3.5. Correlation Analysis

#### 3.5.1. Correlation Between Microbial Diversity and Volatile Flavor Components

**Figure 6:**
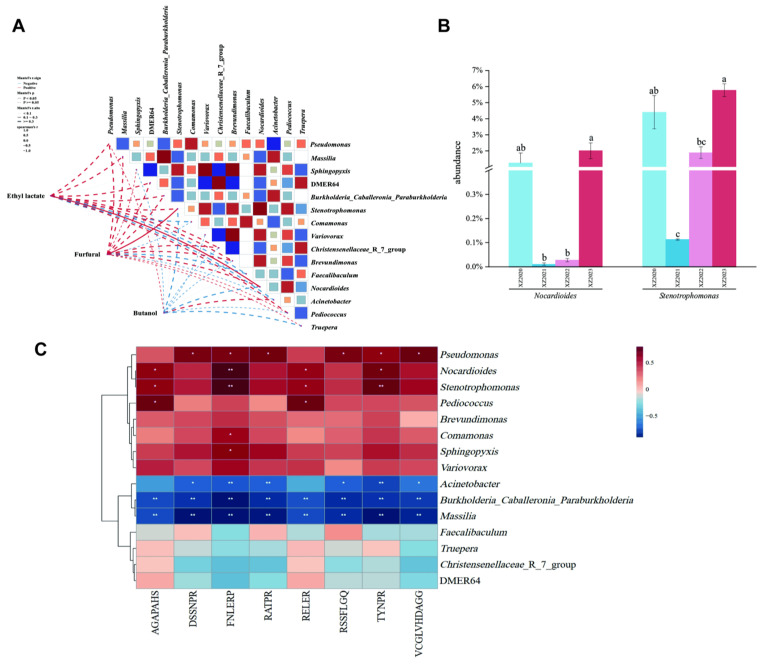
Microbe and flavor substance correlation analysis of *Xiangzao*. (**A**) Correlation analysis between flavor compounds and bacterial species. (**B**) Core species abundance change trend chart. (**C**) Flavor peptide and strain analysis of the correlation. Note: (a–c) Within the same column of data, identical letters indicate no significant difference (*p* > 0.05), while different letters signify a significant difference (*p* < 0.05).

The top 15 bacterial genera with the highest abundance were selected, and the correlation between bacterial genus abundance and high content of odor molecules was further discussed. The degree of correlation between microorganisms and three odor molecules (ethyl lactate, furfural, and n-butyl alcohol) was explored by correlation scores using the Pearson method (Figure 6A). It was found that although there were diversity correlations between bacterial genus abundance and different odor molecules, ethyl lactate was significantly positively correlated with *Nocardioides* [39]. The correlation between furfural and *Stenotrophomonas* was also a positive significant correlation that had also been reported in the correlation between the two and olefin and other volatile flavor compounds [40,41]. However, the correlation of n-butanol with bacterial genera did not show a significant positive or negative degree. In addition, compounds produced by bacterial metabolism such as propionic acid, benzaldehyde, and butyric acid were also found to have negative or zero correlation with *Nocardioides*, *Oxalobacter,* and *Pseudomonas*.

In addition, the relative abundance of two genera, *Nocardioides* and *Stenotrophomonas,* was found to show a similar trend in all fermentation samples (Figure 6B). There was a sharp decrease from XZ-4Y to XZ-3Y, with a significant decrease in *Stenotrophomonas* (*p* < 0.05). From XZ-2Y to XZ-1Y, two bacteria genera showed an upward trend with significant differences (*p* < 0.05). Moreover, in the XZ-1Y, the bacteria of both genera had the highest relative abundance among all fermentation samples. Environmental changes at this stage may have caused a reduction in the relative abundance of these two kinds of bacteria. We also observed the rise in the relative abundance of the two kinds of bacteria. The results showed that the trend of microbial changes in the fermentation process was closely related to the fermentation conditions. By optimizing fermentation conditions, we can effectively control the microbial growth and metabolism that would improve the quality and flavor of the fermented product.

#### 3.5.2. Correlation Between Microbial Diversity and Flavor-Presenting Peptides

The top 15 bacterial genera with the highest abundance were selected, and the correlation between the abundance of bacterial genera and the eight identified umami peptides was analyzed by Spearman’s method (Figure 6C). The results showed that *Pseudomonas* was positively correlated with the abundance of most umami peptides, followed by *Nocardioides* and *Stenotrophomonas* in microbial genera with relatively high abundance. These bacteria have a wide range of environmental ecological distribution and strong protein hydrolysis ability, and they can secrete a variety of proteases or peptidases. The results in Figure 6C also showed that *Burkholderia, Caballeronia, Paraburkholderia,* and *Massilia* were significantly negatively correlated with the abundance of eight umami peptides, followed by *Acinetobacter*. This was attributed to the type of substrate on which they grew.

## 4. Discussion

The acid in Huangjiu is produced by yeast metabolism. The increase in alcohol content during the late fermentation stage inhibits yeast activity and slows down the reproduction of yeast [42]. Yeast still exists in the dregs after pressing; thus, spices and salt are added to make the plasmic walls of the yeast separated and inactive, which fully reflects the acidity, ester fragrance, and aroma of aging [43]. Lactic acid [44] was the most prominent organic acid in *Xiangzao,* providing a refreshing sour flavor [45,46]. In general, the total organic acid content increased gradually with the extension of fermentation time. Total nitrogen refers to all nitrogen compounds in the sample, while amino acid nitrogen refers to the content of nitrogen in the form of amino acids. The higher content of amino acid nitrogen represents the fuller degradation of raw protein [47]. Cao et al. [48] found that active enzymes had a positive effect on the content of amino acid nitrogen in wheat qu. Rice is the main source of protein in *Huangjiu*. Rice protein is degraded by enzymes in wheat qu to produce amino acid nitrogen. This study showed that the highest enzyme activity of XZ-1Y was found in *Xiangzao* at the early stage of fermentation, resulting in a higher content of amino acid nitrogen.

Esters accounted for the majority of volatile flavor compounds in *Xiangzao* in terms of type and content. Esters synthesized from short-chain acids usually contribute to fruit aroma, while those synthesized from long-chain acids provide fat [49]. Diethyl succinate had the highest content in *Xiangzao* of XZ-4Y. The second was XZ-1Y, and the lowest content was in XZ-3Y and XZ-2Y, while ethyl acetate had comparable content in the four years of *Xiangzao*. In general, the volatile compounds in *Xiangzao* showed dynamic changes during fermentation, which might be related to the succession of microbial activities; combining the time cost and flavor performance, the XZ-1Y presents the best style. The distribution of flavor peptides revealed that peptides from different fermentation years were predominantly umami peptides, constituting 70.00% to 72.54% of the total. In contrast, bitter peptides were significantly less abundant, ranging from 5.88% to 8.67%. Notably, the XZ-1Y sample exhibited the highest concentration of Xiangtong halogen peptides. Analysis of the interactions of eight umami peptides (RATPR, RELER, FNLERP, TYNPR, DSSNPR, RSSFLGQ, AGAPAHS and VCGLVHDAGG) with the flavor receptors T1R1/T1R3 showed that the umami peptides mainly formed hydrogen bonds and hydrophobic interactions with the receptors, followed by salt–bridge interactions. The analysis of bondomh types revealed that the umami peptides mainly tended to form more hydrogen bonds with the T1R1 receptor, even though their binding energy was slightly lower than that of the T1R3 receptor (Figure S2). This is consistent with the conclusion of Zhang et al. [50] that the T1R1 receptor is the main receptor for the interaction of umami peptides and umami receptors.

By investigating the bacterial genus abundance of odor molecules, it was found that microbial metabolism processes may not only depend on a single odor molecule but may be involved in the metabolism of multiple odor molecules at the same time. For example, aminopeptidases can hydrolyze hydrophobic amino acid residues such as leucine and proline, resulting in a decrease in bitterness. Zhang et al. [51] demonstrated that Pseudomonas had excellent polypeptide degradation ability in batch fermentation of soybean paste. Proteases produced by Pseudomonas aeruginosa are able to hydrolyze wheat gluten protein into active peptides with low molecular weight [52]. Oligotrophomonas can secrete basic serine proteases and efficiently degrade feather proteins and proteins to polypeptides [53]. These results suggest that the production of peptides from P. fragrans is closely related to the activities of microbial extracellular enzymes. For example, Massiliensis mainly secretes oxidases, lipases, amylase, and cellulases, making it closely related to the production of small molecules such as amino acids and lipids [54]. In summary, the complex microbial network during the fermentation process of *Xiangzao* provides diverse pathways for the formation of peptides.

## 5. Conclusions

In this study, the physicochemical indexes, flavor components, peptide distribution, and microbial composition of *Xiangzao* were analyzed to explore the effects of different fermentation years on flavor quality. Physical and chemical analysis showed that organic acids, total nitrogen, and amino acids showed a downward trend during fermentation. The XZ-4Y sample fermented for 4 years had the highest contents of organic acids and fruity components. The XZ-1Y sample fermented for 1 year had the highest total content of flavor substances, as well as the highest abundance and quantity of peptide segments, which were mainly umami peptides. In addition, four unreported potential umami peptides (FATPR, RELER, FNLERP, RSSFLGQ) were also screened. Metagenomics showed that there were significant differences in the composition of *Xiangzao* from different fermentation years. Ethyl lactate and furfural were significantly positively correlated with the abundance of *Nocardioides* and *Stenotrophomonas*, respectively. *Pseudomonas* was positively correlated with the abundance of umami peptides identified. These results provide insight into the formation of flavor compounds and flavor-forming peptides, as well as an important reference for the flavor stability and quality control of *Xiangzao*.

## Data Availability

The original contributions presented in the study are included in the article/Supplementary Material, and further inquiries can be directed to the corresponding author.

## Supplementary Materials

The following supporting information can be downloaded at https://www.mdpi.com/article/10.3390/foods13233931/s1. Figure S1: Volcano plot of different peptides in *Xiangzao* samples from different years. Figure S2: Heat map of the bonding type between *Xiangzao* umami peptide and T1R1/T1R3 receptors. Table S1: Physicochemical indices of *Xiangzao* in different fermentation years (μg/kg dry matter). Table S2: Volatile flavor compounds of *Xiangzao* in different fermentation years (μg/kg dry matter). Table S3 Binding energy between *Xiangzao* umami peptide and T1R1/T1R3 receptors. Table S4: Species biomarker data of bacterial microbial community structure in *Xiangzao*. Table S5: Species biomarker data of fungi microbial community structure in *Xiangzao*.
