# Supplementary material for: The Microbial Diversity and Flavor Metabolism Regulation of Xiangzao During Different Natural Fermentation Time Periods
Source: Foods. 2024 Dec 5;13(23):3931. doi: 10.3390/foods13233931 (PMC11641313; doi:10.3390/foods13233931)
Supplement: Supplementary file 1 [file foods-13-03931-s001.zip › foods-3332589-supplementary.pdf]

The Microbial Diversity and Flavor Metabolism Regulation of *Xiangzao* During Different Natural Fermentation Time Periods

Table S1. Physicochemical indices of *Xiangzao* in different fermentation years (μg/kg dry matter)

	Indicators	XZ-Y1	XZ-Y2	XZ-Y3	XZ-Y4
1	pH	3.41±0.01^{a}	3.48±0.01^{a}	3.56±0.01^{a}	3.42±0.01^{a}
2	Total acid	55.84±0.50^{b}	39.21±0.37^{b}	47.21±0.70^{c}	53.95±0.16^{a}
3	Total organic acids	42.33±3.28^{c}	31.69±0.14^{a}	31.17±0.25^{a}	42.00±0.14^{a}
4	Lactic acid	33.48±3.16^{a}	24.80±0.09^{b}	22.30±0.09^{b}	29.13±0.02^{c}
5	Acetic acid	4.38±0.08^{a}	2.22±0.18^{c}	2.35±0.04^{a}	4.47±0.22^{b}
6	Propionic acid	2.09±0.05^{b}	2.46±0.04^{b}	4.26±0.10^{c}	5.16±0.09^{a}
7	Pyruvic acid	0.16±0.01^{d}	0.09±0^{c}	0.03±0^{a}	0.05±0^{c}
8	Isovaleric acid	0.05±0.01^{b}	0.11±0.09^{c}	0.22±0.03^{a}	0.19±0^{b}
9	Succinic acid	0.29±0.01^{c}	0.42±0^{b}	0.63±0.01^{a}	0.63±0.07^{a}
10	Tartaric acid	1.63±0.02^{a}	1.25±0.01^{b}	0.9±0.16^{c}	1.52±0.07^{a}
11	Oxalic acid	0.23±0.01^{c}	0.25±0.02^{c}	0.41±0.03^{a}	0.83±0.10^{a}
12	Citric acid	0.01±0^{b}	0.03±0^{a}	0.03±0^{a}	0±0^{c}
13	Amino acid nitrogen	5.47±0.10^{a}	3.34±0.09^{b}	3.96±0.09^{c}	4.38±0.09^{a}
14	Total nitrogen	565.10±12.13^{c}	419.53±1.87^{c}	438.46±5.49^{a}	429.60±6.00^{b}

Table S2. Volatile flavor compounds of *Xiangzao* in different fermentation years (μg/kg dry matter)

	Class of Compounds	XZ-Y4	XZ-Y3	XZ-Y2	XZ-Y1
1	Ethyl acetate	19.57±1.76[a]	17.25±2.79[a]	17.55±2.73[a]	19.73±4.33[a]
2	Isobutyl acetate	0.02±0[b]	0.02±0.01[b]	0.02±0[b]	0.04±0.01[a]
3	Isoamyl butyrate	0.02±0.01[a]	0.02±0[a]	0.01±0[a]	0.02±0[a]
4	Ethyl lactate	4±0.17[b]	2.08±0.15[d]	2.91±0.32[c]	5.83±0.08[a]
5	2-hydroxy-4-methylvalerate ethyl ester	34.64±4.92[a]	14.98±0.78[c]	10.74±1.87[c]	24.6±1.09b
6	Ethyl benzoate	0±0[a]	0±0[a]	0±0[a]	0±0a
7	Diethyl succinate	31.31±4.19[a]	24.97±1.38[b]	22.11±2.71[b]	22.18±0.36[b]
8	Ethyl phenylacetate	0.19±0.02[b]	0.27±0.01[a]	0.15±0.06[b]	0.08±0.01[c]
9	Ethyl 3-phenylpropionate	0±0[a]	0±0[a]	0±0[a]	0±0[a]
10	Ethyl isobutyrate	0.05±0.02[a]	0.04±0.02[a]	0.02±0.01[a]	0.02±0[a]
11	Ethyl butyrate	0.06±0.01[a]	0.05±0.02a	0.04±0.01a	0.07±0.02a
12	Ethyl isovalerate	0.08±0.01[a]	0.06±0.03[a]	0.05±0.01[a]	0.06±0.01[a]
13	Isoamyl acetate	0.08±0[b]	0.07±0.02[b]	0.07±0.01[b]	0.11±0.01[a]
14	Ethyl valerate	0.01±0[b]	0.01±0b	0.01±0b	0.02±0a
15	Ethyl caproate	0.06±0.01[a]	0.06±0.01[a]	0.04±0.01[b]	0.07±0.01[a]
16	Ethyl octanoate	0.01±0[a]	0.01±0[a]	0.01±0[a]	0.01±0[a]
17	Ethyl niacin	0.07±0.02[b]	0.1±0.02[a]	0.09±0.01[a]	0.06±0[b]
18	Phenethyl acetate	0.13±0.03[a]	0.12±0.01[b]	0.1±0.02[b]	0.08±0.01[b]
19	Propionyl nonolactone	0.18±0.07[b]	0.4±0.03[b]	0.61±0.24[b]	2.27±0.37[a]
20	Ethyl cinnamate	0±0[a]	0±0[a]	0±0[a]	0±0[a]
1	Acetaldehyde	0.75±0.09[a]	0.74±0.05[a]	0.73±0.11[a]	0.73±0.08[a]
2	Isovaleraldehyde	1.27±0.08[a]	1.36±0.11[a]	1.02±0.1[b]	1.17±0.06[b]
3	Furfural	18.12±0.82[b]	6.91±0.74[c]	11.85±5.08[c]	21.82±4.4[a]

	Class of Compounds	XZ-Y4	XZ-Y3	XZ-Y2	XZ-Y1
4	Benzaldehyde	1.18±0.05^{b}	1.24±0.14^{b}	1.2±0.12^{b}	2.39±0.26^{a}
5	Phenylacetaldehyde	0.13±0.02^{b}	0.15±0.03^{b}	0.12±0.04^{b}	0.24±0.05^{a}
6	Acetophenone	0.05±0.01^{b}	0.06±0^{a}	0.04±0.01^{b}	0.04±0.01^{b}
7	5-methyl-2-thiophenaldehyde	0±0^{a}	0±0^{a}	0±0^{a}	0±0^{a}
8	Hexanal	0.05±0.01^{b}	0.03±0.01^{b}	0.04±0.01^{b}	0.09±0.01^{a}
9	Vanillin	0.2±0.05^{c}	0.15±0.01^{c}	0.31±0.09^{b}	0.55±0.11^{a}
10	Nonenal	0.88±0.05^{b}	1.02±0.08^{b}	0.87±0.02^{b}	1.09±0.13^{a}
1	N-hexanol	0.25±0.01^{c}	0.23±0.02^{c}	0.36±0.05^{b}	0.62±0.05^{a}
2	N-butanol	0.01±0.01^{a}	0.01±0.01^{a}	0.02±0.01^{a}	0.02±0.01^{a}
3	Heptanol	0.1±0^{c}	0.09±0^{c}	0.11±0.01^{b}	0.15±0.02^{a}
4	Benzyl alcohol	0.31±0.03^{a}	0.27±0.03^{a}	0.25±0.03^{a}	0.28±0.01^{a}
5	1-octene-3-ol	0.05±0^{b}	0.06±0^{b}	0.06±0.01^{b}	0.13±0.01^{a}
1	Caproic acid	0.41±0.06^{c}	0.34±0.04c	0.48±0.09^{b}	1.22±0.07^{a}
2	Phenol b	0.08±0.03^{a}	0.09±0.01^{a}	0.08±0.03^{a}	0.05±0.01^{a}
3	Nonanoic acid	0±0^{a}	0±0^{a}	0±0^{a}	0.01±0^{a}
4	Guaiacol	0.02±0.01^{a}	0.02±0^{a}	0.02±0.01^{a}	0.02±0^{a}
5	4-ethyl phenol	0.02±0.01^{b}	0±0^{c}	0.01±0.01^{c}	0.06±0.01^{a}
1	2-Propionyl furan	0.11±0.02^{a}	0.11±0^{a}	0.07±0.03^{b}	0.03±0^{c}

Note: Different superscript letters on the same line indicate significant differences ($P<0.05$).

Table S3. Binding energy between *Xiangzao* umami peptide and T1R1/T1R3 receptors

	Peptide sequence	Molecular weight	T1R1 (kcal/mol)	T1R3 (kcal/mol)	Sources	Abundance Proportion (%)
1	RATPR	599.69	9.53	9.58	A4FKX2	2.234
2	RELER	701.78	8.87	9.80	A4FE38	2.172
3	FNLERP	774.87	8.87	9.11	A0A6N1X647	2.971
4	TYNPR	649.70	8.36	9.48	P07728	1.660
5	RSSFLGQ	793.87	8.12	9.18	Q5QLS8	3.230
6	DSSNPR	674.66	8.12	9.39	A0A0P0V8W3	2.501
7	AGAPAHS	609.64	8.09	9.74	A0A6N1X0J4	2.524
8	VCGLVHDAGG	927.04	7.99	8.91	A4FGD8	1.361
9	DNENRP	743.73	7.90	8.77	A0A3B6C3H3	1.997
10	LQHTGF	701.78	7.75	9.69	A0A6N1X490	1.746
11	ENTHNGA	741.71	7.70	9.54	A4FQJ5	1.412
12	HAMLDQGV	869.99	7.47	8.18	UPI00070FC8B0	1.329
13	ADTYNPR	835.87	7.20	8.96	P07728	4.668
14	ITQGRAR	800.91	7.10	9.36	P07728	2.105
15	VFHGGSGS	746.77	6.74	10.20	A0A3G2S1Y2	1.316
16	NVNAHSL	753.81	6.60	9.09	A0A0H2MPV4	1.215
17	VIEPRGL	782.93	6.50	8.95	A0A0P0V8F8	14.15
18	VQMSAVK	761.93	6.27	7.45	P07728	1.139
19	PHYTNG	687.71	6.19	10.02	P07728	3.674
20	VYITQGR	835.95	5.75	9.24	P07728	1.163
21	RADTYNPR	992.06	5.31	8.58	P07728	1.571
22	AVLHDGTP	808.89	4.49	8.56	WP_083684893. 1	1.090
23	ITQGRARVQ	1028.18	4.17	8.62	P07728	1.866
24	VQVVNNNGK	971.08	4.15	7.37	P07728	1.445
25	VIEPRGLL	896.09	2.67	7.77	A0A0P0V8F8	11.65

Table S4. Species biomarker data of bacterial microbial community structure in *Xiangzao*

species	XZ-4Y1	XZ-4Y2	XZ-4Y3	XZ-3Y1	XZ-3Y2	XZ-3Y3	XZ-2Y1	XZ-2Y2	XZ-2Y3	XZ-1Y1	XZ-1Y2	XZ-1Y3
Pseudomonas_stutzeri	0.347	0.614	0.596	0.006	0.007	0.005	0.366	0.626	0.712	0.578	0.573	0.697
Massilia_putida	0.018	0.002	0.015	0.186	0.320	0.170	0.003	0.007	0.000	0.000	0.000	0.000
Sphingopyxis_nepalensis	0.301	0.044	0.056	0.001	0.000	0.001	0.033	0.018	0.068	0.066	0.064	0.055
Rikenellaceae_bacterium_DTU002	0.000	0.000	0.000	0.231	0.012	0.247	0.064	0.000	0.000	0.003	0.000	0.000
Stenotrophomonas_geniculata	0.023	0.053	0.055	0.001	0.001	0.001	0.015	0.015	0.026	0.064	0.057	0.051
Comamonas	0.005	0.016	0.015	0.001	0.001	0.001	0.008	0.284	0.002	0.009	0.011	0.008
Variovorax_paradoxus	0.172	0.021	0.032	0.001	0.001	0.001	0.000	0.002	0.002	0.032	0.040	0.018
Trinickia_soli	0.007	0.001	0.006	0.068	0.087	0.060	0.001	0.003	0.000	0.000	0.000	0.000
Pseudomonas_fluorescens	0.000	0.000	0.000	0.000	0.000	0.000	0.225	0.000	0.000	0.001	0.001	0.000
Massilia_tieshanensis	0.001	0.000	0.001	0.013	0.146	0.012	0.000	0.001	0.000	0.000	0.000	0.000
Pseudomonas_putida	0.007	0.016	0.019	0.000	0.000	0.000	0.038	0.007	0.034	0.014	0.012	0.012
Brevundimonas_vesicularis	0.025	0.013	0.017	0.001	0.000	0.001	0.021	0.001	0.005	0.015	0.016	0.011
unclassified_Gottschalkia	0.000	0.000	0.001	0.051	0.004	0.056	0.006	0.000	0.000	0.002	0.000	0.000
Faecalibaculum_rodentium	0.005	0.000	0.000	0.000	0.000	0.000	0.000	0.000	0.093	0.000	0.000	0.000
Pimelobacter_simplex	0.001	0.013	0.023	0.000	0.000	0.000	0.000	0.000	0.000	0.025	0.025	0.010
Acinetobacter_baumannii	0.040	0.002	0.000	0.002	0.041	0.002	0.000	0.000	0.000	0.000	0.000	0.000
Paraburkholderia_caffeinilytica	0.002	0.000	0.002	0.024	0.033	0.022	0.000	0.001	0.000	0.000	0.000	0.000
Pediococcus_pentosaceus	0.000	0.014	0.001	0.000	0.001	0.001	0.000	0.000	0.000	0.005	0.022	0.034
unclassified_Christensenellaceae_R_7_group	0.000	0.000	0.000	0.027	0.002	0.026	0.021	0.000	0.000	0.001	0.000	0.000
Achromobacter_xylosoxidans	0.001	0.013	0.017	0.000	0.000	0.000	0.000	0.001	0.001	0.015	0.015	0.012
Others	0.045	0.155	0.135	0.382	0.339	0.393	0.193	0.034	0.053	0.159	0.158	0.089
Unknown	0.001	0.024	0.009	0.005	0.003	0.002	0.002	0.001	0.000	0.011	0.006	0.002

Table S5. Species biomarker data of fungi microbial community structure in *Xiangzao*

species	XZ2001	XZ2002	XZ2003	XZ2101	XZ2102	XZ2103	XZ2201	XZ2202	XZ2203	XZ2301	XZ2302	XZ2303
Diutina_catenulata	0.143	0.129	0.139	0.143	0.139	0.129	0.181	0.165	0.172	0.159	0.150	0.134
Thermomyces_lanuginosus	0.017	0.026	0.020	0.017	0.020	0.026	0.038	0.045	0.034	0.027	0.027	0.028
Aspergillus_heterocaryoticus	0.007	0.007	0.007	0.007	0.007	0.007	0.030	0.033	0.026	0.018	0.018	0.017
Malassezia_restricta	0.010	0.016	0.011	0.010	0.011	0.016	0.000	0.000	0.000	0.211	0.208	0.222
Trichosporon_coremiiforme	0.103	0.100	0.107	0.103	0.107	0.100	0.037	0.045	0.053	0.010	0.010	0.011
Alternaria_destruens	0.029	0.026	0.025	0.029	0.025	0.026	0.057	0.077	0.082	0.035	0.029	0.030
Rhinocladium_lesnei	0.106	0.092	0.092	0.106	0.092	0.092	0.025	0.023	0.024	0.000	0.000	0.000
unclassified_Fungi	0.018	0.019	0.019	0.018	0.019	0.019	0.038	0.025	0.026	0.015	0.014	0.013
Cladosporium_austroafricanum	0.003	0.004	0.004	0.003	0.004	0.004	0.039	0.032	0.035	0.036	0.037	0.036
Humicola_homopilata	0.045	0.054	0.051	0.045	0.051	0.054	0.017	0.016	0.017	0.000	0.000	0.000
Olpidium_brassicae	0.000	0.000	0.000	0.000	0.000	0.000	0.000	0.000	0.000	0.026	0.025	0.025
unclassified_Ascomycota	0.000	0.002	0.001	0.000	0.001	0.002	0.038	0.041	0.035	0.012	0.012	0.011
Naganishia_uzbekistanensis	0.004	0.004	0.003	0.004	0.003	0.004	0.010	0.010	0.010	0.018	0.012	0.013
Triadelphia_pulvinata	0.013	0.024	0.020	0.013	0.020	0.024	0.000	0.000	0.000	0.000	0.000	0.000
unclassified_Basidiomycota	0.004	0.004	0.003	0.004	0.003	0.004	0.002	0.000	0.000	0.006	0.006	0.006
Apiotrichum_scarabaeorum	0.023	0.019	0.023	0.023	0.023	0.019	0.000	0.000	0.000	0.000	0.000	0.000
Apiotrichum_loubieri	0.048	0.028	0.034	0.048	0.034	0.028	0.005	0.005	0.007	0.000	0.000	0.000
Fusarium_foetens	0.003	0.005	0.003	0.003	0.003	0.005	0.006	0.006	0.006	0.018	0.018	0.017
Aspergillus_fumigatus	0.012	0.016	0.015	0.012	0.015	0.016	0.009	0.005	0.005	0.015	0.016	0.017
Dipodascus_australiensis	0.018	0.017	0.017	0.018	0.017	0.017	0.003	0.000	0.000	0.006	0.006	0.006
Others	0.249	0.262	0.263	0.249	0.263	0.262	0.311	0.291	0.323	0.192	0.206	0.205
unidentified				0.147	0.143		0.153	0.180	0.145	0.209	0.205	0.147

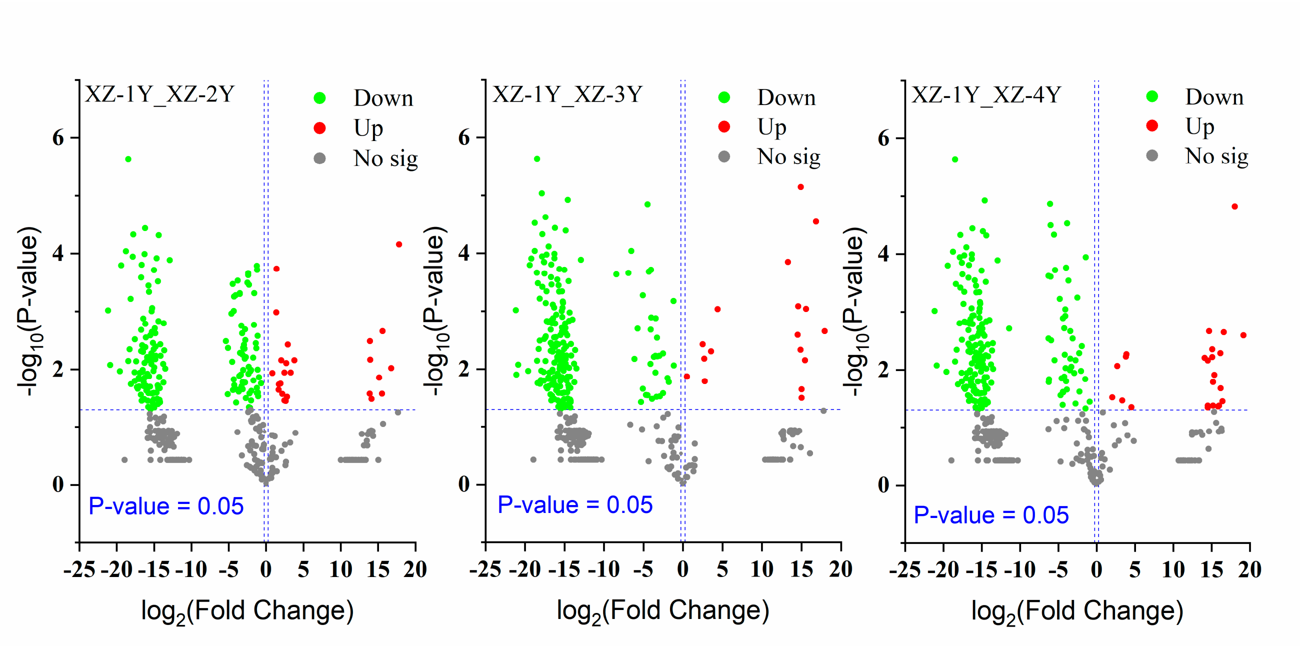

Figure S1. Volcano plot of different peptides in Xiangzao samples from different years

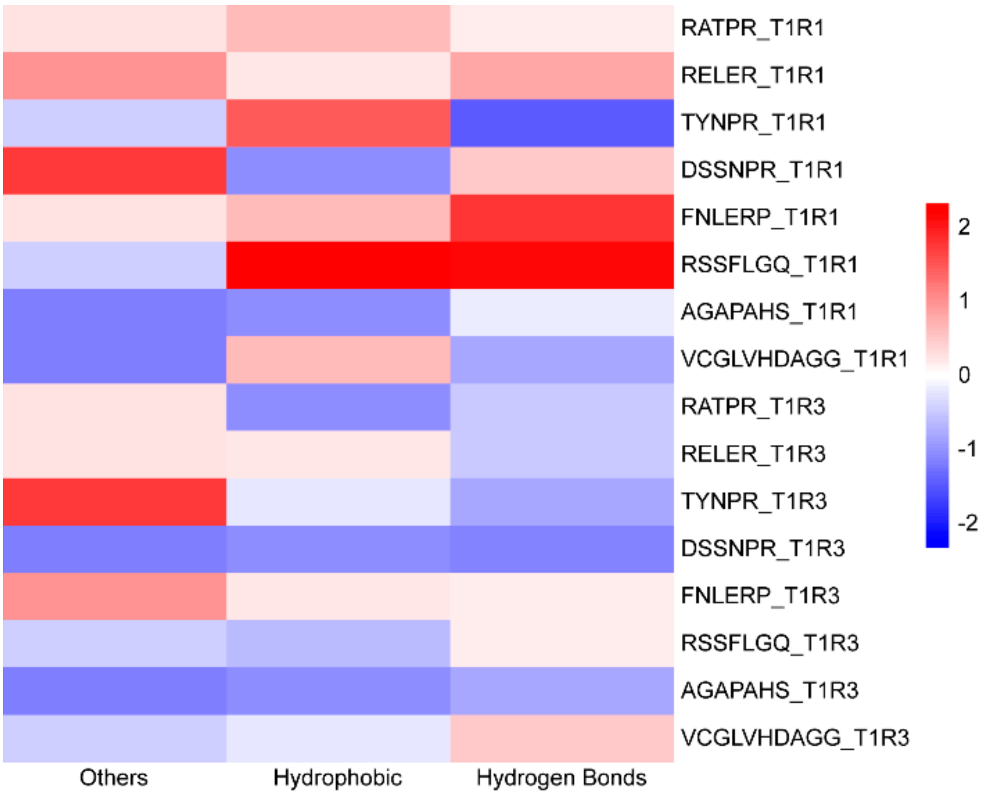

Figure S2. Heat map of the bonding type between *Xiangzao* umami peptide and T1R1/T1R3 receptors
